# Fecal Occult Blood and Fecal Calprotectin as Point-of-Care Markers of Intestinal Morbidity in Ugandan Children with *Schistosoma mansoni* Infection

**DOI:** 10.1371/journal.pntd.0002542

**Published:** 2013-11-14

**Authors:** Amaya L. Bustinduy, José C. Sousa-Figueiredo, Moses Adriko, Martha Betson, Alan Fenwick, Narcis Kabatereine, J. Russell Stothard

**Affiliations:** 1 Department of Parasitology, Liverpool School of Tropical Medicine, Liverpool, United Kingdom; 2 Department of Infectious and Tropical Diseases, London School of Hygiene and Tropical Medicine, London, United Kingdom; 3 Vector Control Division, Ministry of Health, Kampala, Uganda; 4 Department of Production and Population Health, The Royal Veterinary College, Hatfield, United Kingdom; 5 Schistosomiasis Control Initiative, Imperial College of London, London, United Kingdom; Swiss Tropical and Public Health Institute, Switzerland

## Abstract

**Background:**

Calprotectin is a calcium-binding cytoplasmic protein found in neutrophils and increasingly used as a marker of bowel inflammation. Fecal occult blood (FOB) is also a dependable indicator of bowel morbidity. The objective of our study was to determine the applicability of these tests as surrogate markers of *Schistosoma mansoni* intestinal morbidity before and after treatment with praziquantel (PZQ).

**Methods:**

216 children (ages 3–9 years old) from Buliisa District in Lake Albert, Uganda were examined and treated with PZQ at baseline in October 2012 with 211 of them re-examined 24 days later for *S. mansoni* and other soil transmitted helminths (STH). POC calprotectin and FOB assays were performed at both time points on a subset of children. Associations between the test results and infection were analysed by logistic regression.

**Results:**

Fecal calprotectin concentrations of 150–300 µg/g were associated with *S. mansoni* egg patent infection both at baseline and follow up (OR: 12.5 *P* = 0.05; OR: 6.8 *P* = 0.02). FOB had a very strong association with baseline anemia (OR: 9.2 *P* = 0.03) and medium and high egg intensity schistosomiasis at follow up (OR: 6.6 *P* = 0.03; OR: 51.3 *P* = 0.003). Both tests were strongly associated with heavy intensity *S. mansoni* infections. There was a significant decrease in FOB and calprotectin test positivity after PZQ treatment in those children who had egg patent schistosomiasis at baseline.

**Conclusions:**

Both FOB and calprotectin rapid assays were found to correlate positively and strongly with egg patent *S. mansoni* infection with a positive ameloriation response after PZQ treatment indicative of short term reversion of morbidity. Both tests were appropriate for use in the field with excellent operational performance and reliability. Due to its lower-cost which makes its scale-up of use affordable, FOB could be immediately adopted as a monitoring tool for PC campaigns for efficacy evaluation before and after treatment.

## Author Summary

The severity of intestinal schistosomiasis, a disease caused by *Schistosoma mansoni* infection, is likely under-reported in part due to the scarcity of field-appropriate morbidity markers. Downstream potential complications of this disease include anemia, failure to thrive, and chronic multi-organ damage. Point-of-care (POC) tools to monitor intestinal schistosomiasis in low resource settings are urgently needed to better quantify the burden of disease in endemic countries and to gauge the clinical impact of scale-up of preventive PC. For the present study in rural Uganda, fecal occult blood and fecal calprotectin were identified as potential surrogate markers of intestinal morbidity. We tested both POC tests and found that they were both associated with active schistosomiasis as detected by eggs in stool with significant decrease in test positivity after PZQ treatment demonstrating short term morbidity reversion. Calprotectin was a strong indicator of intestinal inflammation, however, owing to its high per-test price makes it difficult to scale-up accordingly. Conversely, fecal occult blood was technically feasible, low-cost and had optimal performance as a morbidity marker, hence we strongly advocate for its immediate inclusion as a monitoring tool for PC programs.

## Introduction

With over 207 million people worldwide infected with schistosomiasis, mostly in sub-Saharan Africa, there is still a pressing need to optimize field-appropriate tools for morbidity staging and monitoring of clinical disease [1]. In recent years there has been a scale-up of schistosomiasis control programs worldwide [2], [3]. However, no single direct morbidity marker has been adopted to monitor the clinical impact of these interventions [4]. Part of the difficulty is due to the variability of schistosomiasis-related clinical manifestations that are often non-specific and species dependent. This holds particularly true for intestinal schistosomiasis, mainly caused by two different species of the genus *Schistosoma*: *S. japonicum* and *S. mansoni*, in which the clinical diagnostic gold-standard for bowel morbidity is a colonoscopy. This has limited applicability in resource limited settings owing to logistic constraints [5]. Hence it is important to find suitable low-cost reliable proxy markers of bowel morbidity that can aid in measuring the clinical impact of control programs.

Intestinal schistosomiasis is an inflammatory disease in which the observable pathology derives from the local inflammatory host response to the egg-entrapment inside the intestinal mucosa and submucosa [5]. It can manifest with a wide range of symptoms depending on the extent of the inflammation and friability of the mucosa, ranging from intermittent abdominal pain to overt acute dysentery. By performing colonoscopy studies, colorectal schistosomiasis has been consistently associated with findings of intestinal polyps and pseudopolyps that can present as blood-per-rectum in affected individuals [6].

Across community-based surveys worldwide, detection of intestinal blood loss in schistosomiasis-endemic areas has revealed a positive association between egg-patent infection and presence of blood in stools [7]–[9]. However, the majority of these studies used guaiac-based fecal occult blood (FOB) tests that are known to be less sensitive than the FOB immunochemical assays now widely used for colorectal cancer detection [10], [11]. FOB has also been used for identifying blood loss in parasitic enteric infections with hookworm, *Trichuris trichiura or Entamoeba histolytica* [12]–[14]. For intestinal schistosomiasis a longitudinal FOB study in Ugandan children treated with praziquantel (PZQ) showed a strong correlation with infection before and one year after intervention, highlighting its potential use as a morbidity marker across field surveys [15], [16]. Shorter term disease dynamics need investigation, in particular during the standard cure rate monitoring period at 24 days post PZQ treatment [17].

Calprotectin is a neutrophil cytoplasmic calcium-binding protein that is also found in monocytes and early stage macrophages. Its degranulation inside the intestinal lumen occurs as a response to local inflammation [18]. Detection of calprotectin in stool is currently used across gastroenterology practices to aid diagnostically in distinguishing between inflammatory bowel disease and other non-inflammatory ailments, thereby decreasing the number of unnecessary endoscopies performed. It is also used as a validated marker for disease activity and response to treatment [19]–[21]. Its use in enteric infections is gaining recognition, particularly as a correlative marker for clinical severity in infectious diarrhea from both viral and bacterial etiologies [22]. Based on its potential as a surrogate inflammatory maker, we had previously hypothesized that fecal calprotectin could be elevated in schistosomiasis-associated intestinal pathology. However upon field-testing of an enzyme-linked immunosorbent assay, we found no correlation with *S. mansoni* infection [16]. The study was limited by the complexity and low sensitivity of the ELISA protocol and high minimum calprotectin value detectable in stool. However the low *S. mansoni* prevalence in the area may have also affected the association with the study outcome [16]. With the recent commercial availability of a new POC calprotectin assay based upon immunochromatographic dipsticks, this new test format has greater sensitisation and detection range of calprotectin found in feces.

The primary objective of the present study was an investigation of point-of-care (POC) chromatographic FOB detection and fecal calprotectin with *S. mansoni* infection status as a proxy for intestinal morbidity. Secondary objectives were to ascertain their association with less specific schistosomiasis downstream manifestations such as anemia and to investigate the short-term changes 24 days after PZQ treatment.

## Methods

### Ethics statement

Ethical approval was obtained from both the Liverpool School of Tropical Medicine and the Uganda National Council of Science at the Ministry of Health. For all participants information sheets describing the study were delivered before recruitment. Informed written consent was obtained from the children's parents/guardians and verbal assent from the subjects when possible.

### Study population and study setting

**Figure 1:**
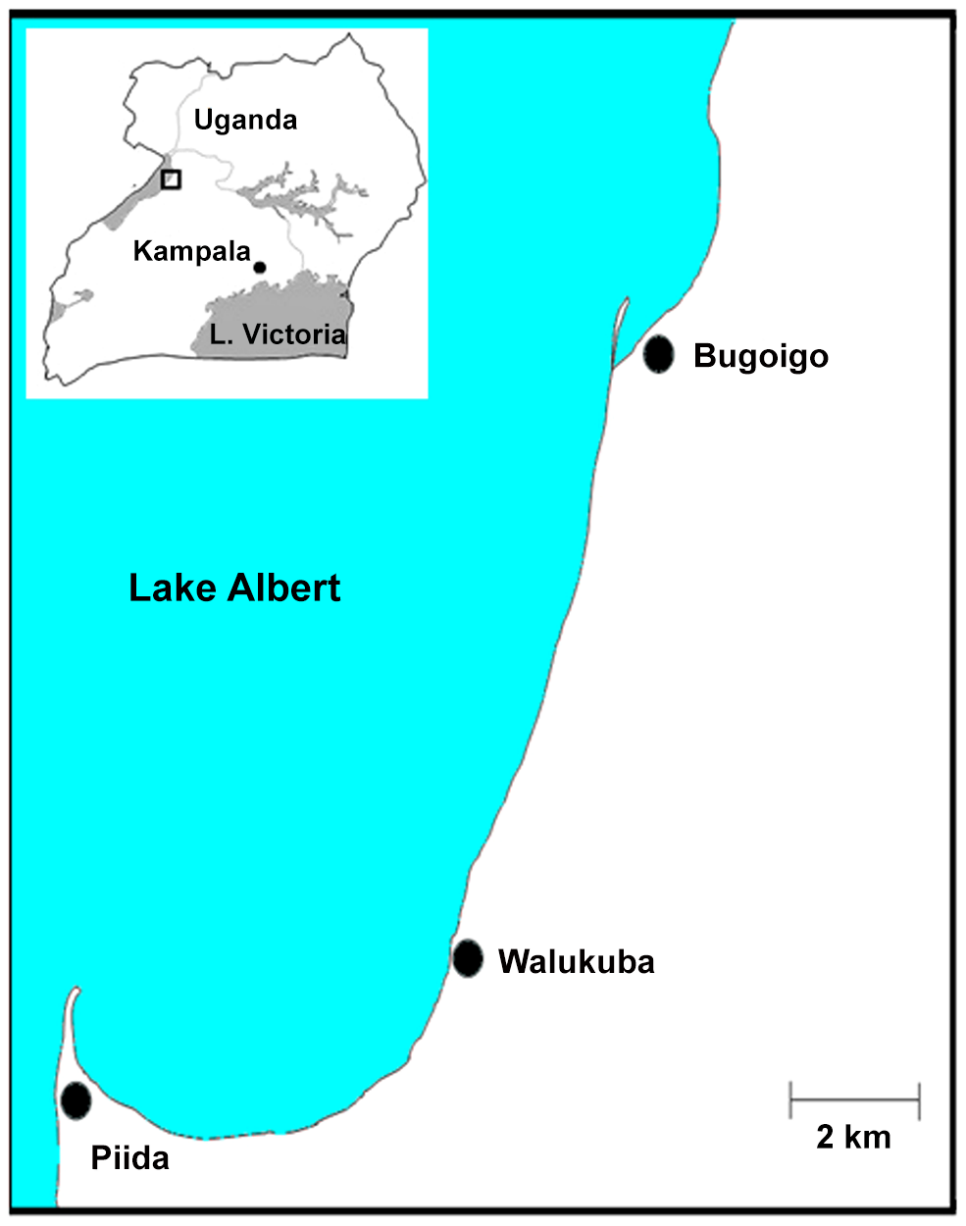
Map of the study villages in Buliisa district, Lake Albert, Uganda.

The study was conducted as an exit investigation from a large longitudinal cohort study based in two villages on the Lake Albert shoreline in Buliisa district, Uganda (**Figure 1**) during the month of October 2012 with a follow up survey 24 days later in November 2012. Children had previously been recruited for a longitudinal study targeting young children with schistosomiasis that started in 2009 (Schistosomiasis in Infants and Mothers (SIMI) cohort) [23]. A total of 216 children were initially surveyed with 211 of them providing stool and urine at follow-up. Only those children with two stool samples at both time points were included in the analysis.

### Parasitological detection and PZQ treatment

Children provided two stool samples on two consecutive days and one urine sample. Duplicate stool smears were prepared from each sample applying the thick Kato-Katz technique for the detection of *S. mansoni* and other soil-transmitted helminths. [24]. Means of eggs per gram of stool (epg) were subsequently calculated from the egg count slide results. Three infection categories according to WHO standards were considered for intestinal schistosomiasis [25]: (1–99 epg) or ‘Light’, (100–399) epg or ‘Medium’ and (>400 epg) or ‘Heavy’. A cathodic circulating antigen (CCA) assay was also employed to detect the presence of *S. mansoni* antigen in urine using a commercially available immuno-chromatographic dipstick (Rapid Medical Diagnostics, Pretoria, South Africa) [26]. All children after providing stool, were treated with PZQ at standard dosing of 40 mg/kg regardless of parasitological results.

### Point-of-care tests: Fecal occult blood, fecal calprotectin, hemoglobin

All children provided a small finger-prick sample (0.5 ml) of blood for estimation of hemoglobin levels by Hemocue (Ängelholm, Sweden). Anemia was then defined according to age-dependent cut-off values [27]. An antigen detection rapid diagnostic test for *Pf*/non-*Pf* malaria was also carried out (Standard Diagnostics, Kyonggi-do, Korea). Children found to be positive were treated according to standard national treatment guidelines.

A simple chromatographic test was employed for FOB detection (Mission Test®, Acon Laboratories, San Diego, CA), following the manufacturer's instructions. Briefly a small amount of feces was homogenised in a liquid buffer after collection. Two drops of stool suspension were applied to a test cassette and results were visually read after five minutes and categorized as negative (−), trace (+/−) and positive (+).

A quantitative point-of–care chromatographic immunoassay was used for the detection of fecal calprotectin (Quantum Blue®, Alpha laboratories, Hampshire, UK), according to the manufacturer's instructions. Two drops of homogenised stool were applied inside a plastic cassette that was then inserted into a portable electronic reader that quantified the intensity of the control and test reactions to express a numerical value. The cassette reader was calibrated for each batch of tests. Cut-off values were based on recent clinical consensus [28]. Values of calprotectin >50 µg/g were considered positive [19]. Calprotectin values were further categorised based on findings from clinical gastroenterology studies [20], [28]. Calprotectin values >160 µg/g are considered within the range of severe intestinal inflammation as seen in inflammatory bowel disease [28]. Guided by this findings, we then created sub-categories to explore the relationship of different inflammatory cut-offs with schistosomiasis; ‘Low’ (51–149 µg/g), ‘Medium’ (150–299 µg/g) and ‘High’ (>300 µg/g), for subsequent statistical analysis.

### Data entry and analysis

All data was entered into electronic format using EpiData® (The EpiData Association Odense, Denmark) and later analysed using the R statistical package version 2.14.1 (The R Foundation for Statistical Computing, Vienna, Austria) and Microsoft Excel (Redmond, WA, US). Arithmetic and geometric mean of Williams (GM_W_) were calculated for *S. mansoni* infection intensity. 95% confidence intervals (CI) were calculated for percentages by applying the exact method.

Primary endpoints of interest were FOB and calprotectin positivity and their association with egg-patent and CCA positive *S. mansoni* infection status. After initial exploratory bivariate analysis, multivariable logistic regression modelling was carried out using the binomial variables constructed for FOB and calprotectin as the outcome variables and adjusting for possible confounding variables such as malaria, anemia, sex, age and village location. A separate analysis was also performed for different calprotectin intensity values. Odds ratios (OR) and 95% CI and *P*-values were calculated as measures of association between each variable and the outcomes. *P*-values<0.05 were considered statistically significant.

## Results

In total, 52.5% of the children surveyed were females. The mean age was 5.9 with a range (3–9). Further age categories were created, distinguishing between school age children (6–9 years old) and preschool age children (3–5 years old).

General characteristics of the study population are summarized in **Table 1**. 159 children (84.1%) of the initial 189 with complete records at baseline had calprotectin results at the 24 days follow-up survey. For FOB, 171 children (90%) were followed up out of the 190 initially tested at baseline. Due to the low prevalence of other helminthic STH infections (<0.5%), these were not included in the analysis. Malaria was highly prevalent in the area with over 80% of children infected at baseline on the basis of rapid diagnostic testing.

**Table 1:** Prevalence (in %) and distribution of infection and morbidities at baseline and follow up (24 days) in 216 children surveyed in Buliisa district aged 3–9 years old.

	Intensity	Baseline (95% CI) N = 184	24 days (95% CI) N = 159	*P* value∧
**Fecal Occult Blood**	Any	13.1 (8.6–18.8)	8.1 (4.5–13.3)	0.173
**Fecal Calprotectin**	Any	53.4 (46.0–60.7)	47.1 (39.2–55.2)	0.281
	Low	36.5 (29.6–43.8)	19.4 (13.6–26.5)	**0.0005**
	Medium	7.4 (4.1–12.1)	12.6 (7.8–18.7)	0.146
	High	9.5 (5.7–14.6)	15.1 (9.9–21.6)	0.137
***S. mansoni*** ** (Kato-Katz)** *	Any	63.5 (56.6–70.0)	28.3 (22.0–35.3)	**<0.0001**
	Light	25.1 (19.4–31.5)	17.1 (12.0–23.2)	0.0659
	Medium	16.5 (11.8–22.3)	8.5 (4.9–13.5)	**0.0234**
	Heavy	21.8 (16.4–27.9)	2.6 (0.8–6.1)	**<0.0001**
***S. mansoni*** ** -CCA** **	Any	92.8 (88.1–96.1)	88.4 (82.9–92.7)	0.154
***S.mansoni*** ** Arithmetic mean (epg)**	Any	408 (281.8–535.2)	93.5 (21.2–165.8)	**<0.0001**
***S.mansoni*** ** Geometric mean (epg)**	Any	28.8 (25.4–30.2)	2.5 (−0.8–3.7)	**<0.0001**
**Malaria-RDT**	Any	80.0 (73.5–85.5)	48.9 (41.3–56.4)	**<0.0001**
**Hemoglobin mean(g/dL)**	Any	10.9 (7.0–14.7)	11.3 (9.0–14.0)	**0.006**
**Anemia** +	Any	56.9 (49.5–64.2)	43.8 (36.5–51.4)	**0.016**

184 and 154 children had complete parasitological and POC results at baseline and follow up respectively.

* *S. mansoni* intensities by Kato-Katz: Light: 1–99 epg of stool; Medium: 100–399 egp of stool; Heavy: >400 epg of stool.

** Circulating Cathodic Antigen in urine: includes trace,

+ Anemia as defined by WHO standards:Hb<11 gr/dl ( 0–5 years old) Hb<11.5 (5–12 years old) Hb<7 gr/dl-severe anaemia,

∧ Differences calculated by fisher exact test and Welch t-test.

**Figure 2:**
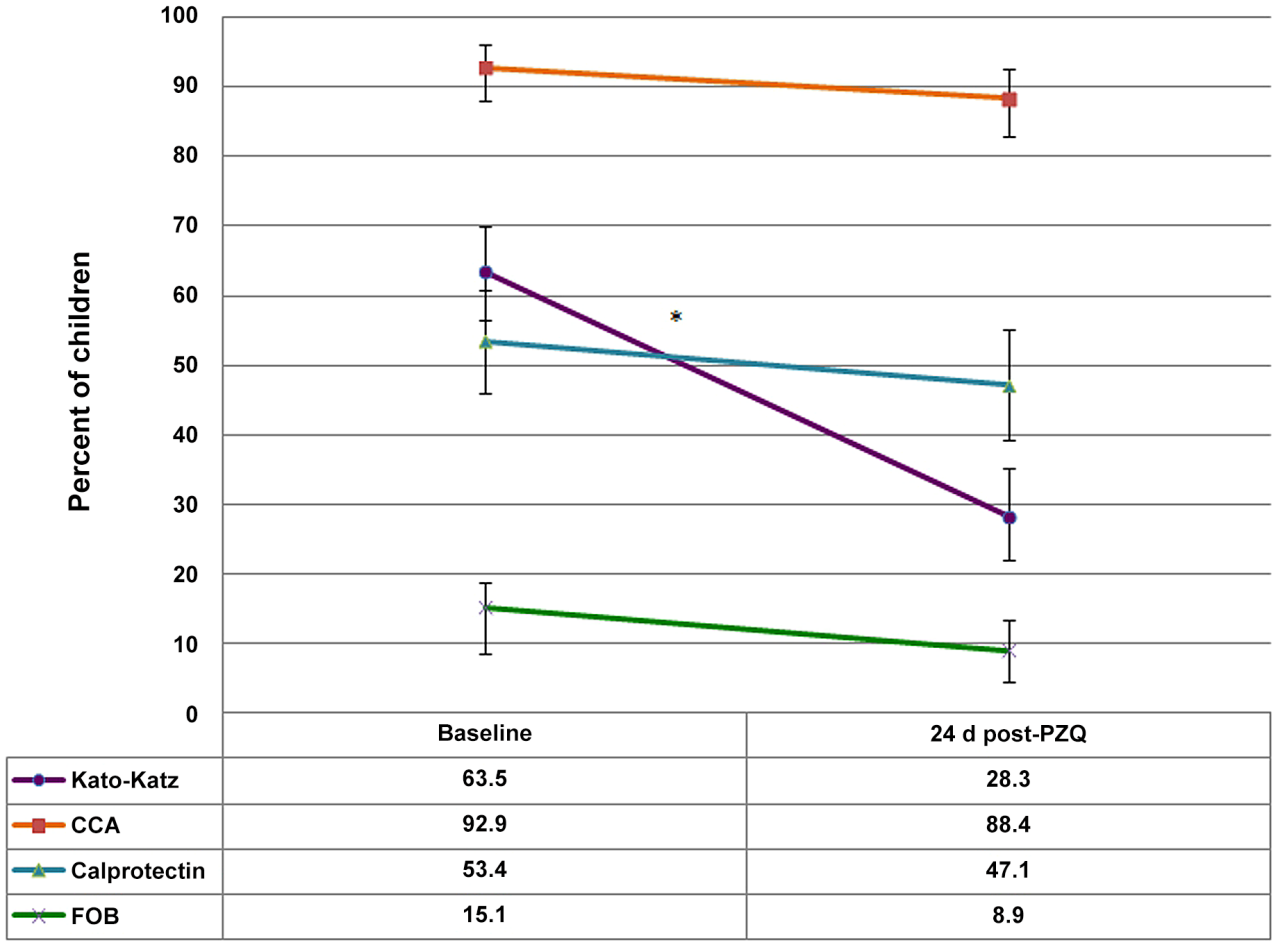
Percent of children positive for the different *S.mansoni* diagnostic tests (Kato-Katz, CCA) as well as stool point-of-care tests (FOB and calprotectin). The decline in Kato-Katz positivity was significant 24 days after treatment.

Overall, there was a high prevalence of egg-patent intestinal schistosomiasis in the area at baseline (63.5%) with almost 22% of children harbouring heavy intensity infections. By CCA test, *S. mansoni* prevalence reached 93% of the children surveyed. As expected, older children (>5 years old) showed a higher prevalence of *S. mansoni* infection than younger children (3–5 years old) (67.2% vs 57.9% with higher infection intensities). *S. mansoni* infection dynamics as assessed by microscopy and CCA testing are shown in **Fig. 2**.

### Association between FOB, calprotectin and *S. mansoni* infection before and after treatment (Table 2 and Figure 3)

**Figure 3:**
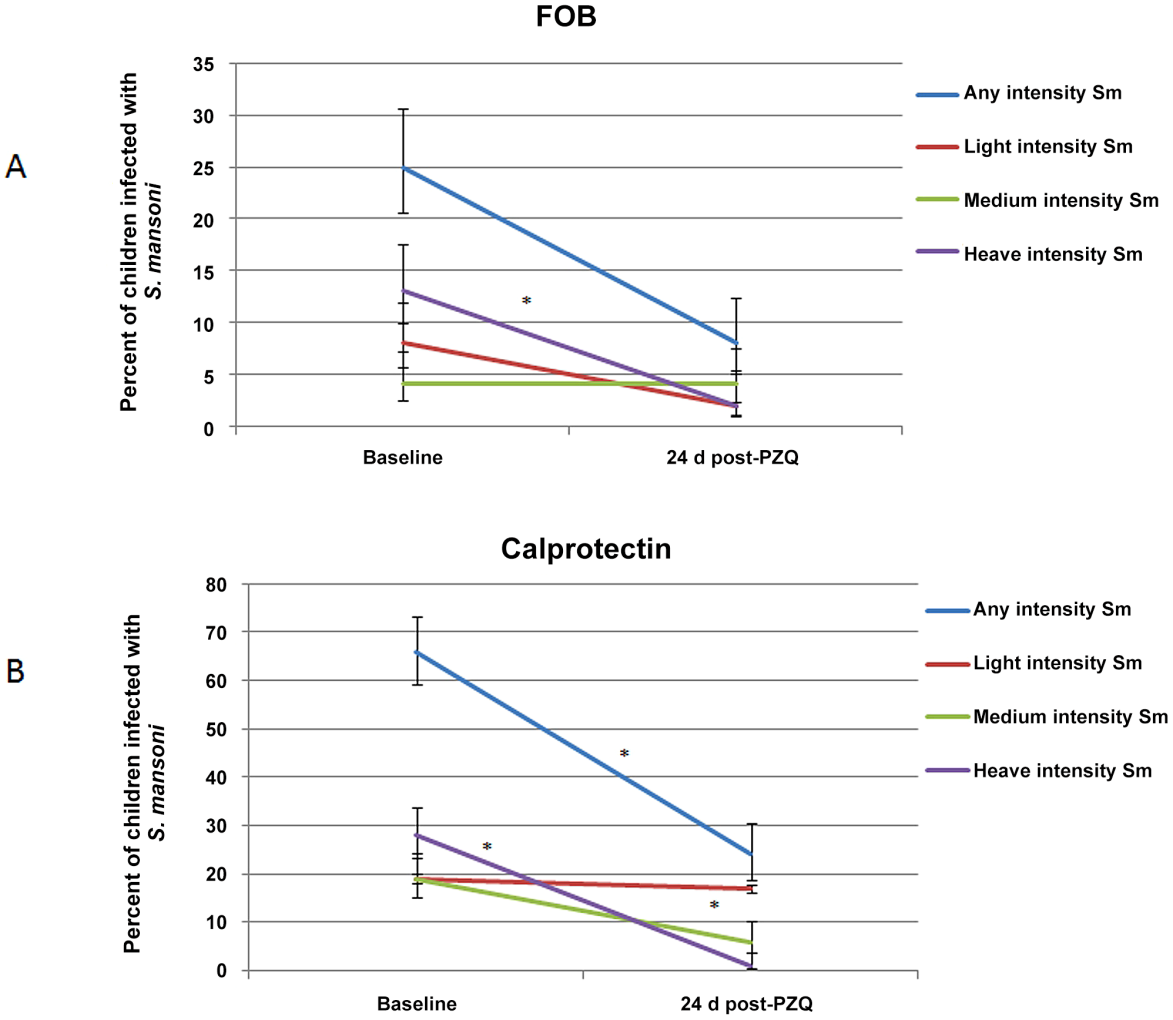
Percent of children egg positive for *S. mansoni* with positive FOB (A) and calprotectin (B) at baseline and 24 days after PZQ treatment. Statistically significant values are indicated ^*^.

Children were stratified by *S. mansoni* infection status at baseline (as assessed by Kato-Katz or CCA test) and the percentage of children positive for FOB and calprotectin in each group was determined both at baseline and 24 days post PZQ treatment (Table 2). The results showed that in the group which was egg patent at baseline, there was a significant decrease in numbers of individuals who were FOB or calprotectin positive 24 days after treatment. This was particularly evident for children with medium intensity infections (for calprotectin *P* = 0.035) and heavy intensity infections (for FOB *P* = 0.007, and calprotectin *P* = <0.0001).

**Table 2:** Total number and percentage (%) of children infected with *S. mansoni* at baseline (n = 117) and at follow up (n = 45) who were positive for FOB and calprotectin before and after treatment.

	FOB		Calprotectin	
Infection status at baseline	Baseline Positive N = 25	Follow up Positive N = 14	Baseline Positive N = 101	Follow up Positive N = 75
***S. mansoni*** ** (Kato-Katz)**				
Any intensity	25 (100)	8 (57.1)+	66 (65.3)	24 (32)∧∧
1–99 epg	8 (32)	2 (14.2)	19 (18.8)	17 (22.6)
100–399 epg	4 (16)	4 (28.5)	19 (18.8)	6 (8)&
>400 epg	13 (52)	2 (14.2)∧	28( 27.7)	1 (1.3)$
***S. mansoni*** ** CCA** *				
Positive	22 (88)	12 (85.7)	14 (13.8)	9 (12)

* Circulating Cathodic Antigen in urine: positives include trace.

+ Significant difference in values Oct–November by Fisher exact test *P* = 0.005;

∧ Significant difference in values Oct–November by Fisher exact test *P* = 0.007;

∧∧ Significant difference in values Oct–November by Fisher exact test *P* = <0.0001;

& Significant difference in values Oct–November by Fisher exact test *P* = 0.035;

$ Significant difference in values Oct–November by Fisher exact test *P* = <0.0001.

### Association between FOB and *S. mansoni* infection (Table 3)

**Table 3:** Fecal Occult Blood logistic regression models at baseline and at 24 days follow up with Adjusted Odds Ratios (AOR) and 95% confident intervals (CI) of the variables included.

	Baseline (N = 190)				Follow up (N = 171)			
	COR* (95% CI)	*P* value	AOR** (95% CI)	P value	COR* (95% CI)	*P* value	AOR (95% CI)	*P* value
School Age (6–9 years)	0.89 (0.3–2.3)	0.83	1.35 (0.5–3.7)	0.55	2.81 (0.7–16.3)	0.15	1.92 (0.4–8.3)	0.36
Female	1.55 (0.6–4.2)	0.39	1.97 (0.7–5.4)	0.19	1.77 (0.5–7.0)	0.40	3.74 (0.9–14.6)	**0.05**
*Schistosoma mansoni* * (Kato-Katz)								
Any intensity	-		-		3.89 (1.2–11.9)	**0.01**	4.28 (1.3–14.0)	**0.01**
1–99 epg	1.51 (0.5–4.1)	0.45	-		0.80 (0.2–3.8)	0.78	1.64 (0.3–9.3)	0.57
100–399 epg	0.90 (0.2–2.9)	0.99	-		5.88 (1.6–22.1)	**0.008**	6.62 (1.1–37.9)	**0.03**
>400 epg	5.23 (1.9–14.0)	**0.0002**	-		8.5 (1.3–56.2)	**0.02**	51.3 (3.7–705.6)	**0.003**
*Schistosoma mansoni* (CCA)	2.85 (1.4–5.9)	**0.004**	2.25 (1.1–4.7)	**0.02**	1.64 (1–2.7)	**0.05**	1.78 (1.0–3.0)	**0.03**
Anemia	13.05 (1.7–99.6)	**0.001**	9.22 (1.2–73.9)	**0.03**	1.91 (0.5–7.6)	0.27	1.39 (0.3–5.3)	0.62
Malaria	1.80 (0.4–10.1)	0.57	1.18 (0.5–2.5)	0.64	0.39 (0.08–1.4)	0.16	0.33 (0.07–1.5)	0.15
Walukuba Village	8.92 (2.5–48.4)	**<0.0001**	7.52 (2.0–27.4)	**0.002**	0.38 (0.09–1.5)	0.17	0.16 (0.03–0.9)	**0.03**

Crude Odds Ratios (COR) are also shown.

* COR = Crude Odd Ratio;

** AOR = Adjusted Odd Ratio;

All models were controlled for age, sex, *Schistosoma mansoni* infection by fecal egg count and urine Cathodic Circulating Antigen (CCA), anemia, malaria infection, fecal calprotectin and village. Reference groups included preschool age, male, uninfected *S. mansoni*, negative CCA, Non-anemia, Non-malaria, Bugoigo village.

*S. mansoni* infection was not included in the baseline model since all FOB positive children were also *S. mansoni* positive.

All children with positive FOB were found to be *S. mansoni* egg positive at baseline and therefore *S. mansoni* was not included in the baseline analysis. After adjusting for potential confounders (age, sex, anemia, malaria) at follow up there was a very strong association between FOB and *S. mansoni* medium and heavy infection (OR: 6.6 *P* = 0.033, OR: 51.3 *P* = 0.003). Children with anemia were very likely to have a positive FOB test result, but only at baseline (OR: 9.2, *P* = 0.036) and girls were more likely to be FOB positive at follow up (OR: 3.7, *P* = 0.057). Residents from Walukuba village were more likely to be FOB positive at baseline (OR: 7.5, *P* = 0.02), however the effect was significantly reversed at follow up (OR: 0.16, *P* = 0.03).

### Association between fecal calprotectin and *S. mansoni* infection (Table 4)

**Table 4:** Fecal calprotectin logistic regression models at baseline and at 24 days follow up with Adjusted Odds Ratios (AOR) and Crude Odds Ratios (CPR) and 95% confident intervals.

	Baseline				Follow up			
	Bivariate analysis		Multivariable analysis		Bivariate analysis		Multivariable analysis	
Any intensity (>50 µg/g)	COR (95% CI)	*P* value	AOR (95% CI)	*P* value	COR (95% CI)	*P* value	AOR (95% CI)	*P* value
3–5 years	1.63 (0.8–3.0)	0.10	2.37 (1.1–5.1)	**0.02**	1.36 (0.7–2.7)	0.41	1.50(0.7–3.2)	0.29
*Schistosoma mansoni^+^*	1.18 (0.6–2.2)	0.65	0.96 (0.4–2.3)	0.93	1.50 (0.7–3.2)	0.28	2.22(0.8–5.6)	0.09
CCA	1.01 (0.2–4.3)	0.99	1.42 (1.0–2.0)	**0.04**	1.16 (0.3–4.3)	0.99	0.89(0.6–1.2)	0.50
FOB	0.91 (0.3–2.3)	0.99	1.59 (0.5–4.7)	0.40	2.75 (0.7–12.8)	0.14	1.79 (0.4–7.4)	0.42
Bugoigo Village	1.94 (1.0–3.6)	**0.02**	2.74 (1.2–6.3)	**0.01**	4.84 (2.3–10.3)	**0.001**	6.06(2.8–13.1)	**<0.0001**
**Light intensity (51–149 µg/g)**								
3–5 years	1.5 (0.8–2.9)	0.25	2.45 (1.0–5.5)	**0.03**	0.52 (0.1–1.4)	0.26	0.51(0.2–1.5)	0.23
*Schistosoma mansoni*	0.92(0.4–1.8)	0.86	0.96 (0.4–2.4)	0.93	1.30(0.4–3.5)	0.63	1.80(0.5–6.2)	0.35
CCA	0.69 (0.1–2.8)	0.75	1.31 (0.9–1.9)	0.12	0.74 (0.2–2.6)	0.77	0.89 (0.5–1.4)	0.65
FOB	0.98(0.3–2.9)	0.99	1.16 (0.3–4.0)	0.80	3.89(0.7–21.2)	0.06	3.1 (0.6–17.1)	0.17
Bugoigo Village	2.23 (1.1–4.5)	**0.01**	2.69 (1.1–6.5)	**0.02**	4.38(1.6–12.7)	**0.001**	4.1(1.5–11.3)	**0.006**
**Medium intensity (150–299 µg/g)**								
3–5 years	1.79 (0.5–6.6)	0.37	4.01 (0.9–17.9)	0.06	3.84 (1.2–13.7)	**0.01**	4.18(1.1–16.0)	**0.03**
*Schistosoma mansoni*	8.09(1.1–357)	**0.03**	12.52(0.99–159.9)	**0.05**	3.15(1.1–9.8)	**0.02**	6.80(1.2–38. 3)	**0.02**
CCA	1.33 (0.7–2.6)	0.38	2.12 (0.7–2.6)	0.16	1.12(0.7–1.7)	0.58	0.76(0.4–1.5)	0.45
FOB	2.65 (0.5–11.5)	0.21	7.56 (0.9–61.1)	0.06	1.03(0.02–11.3)	0.99	0.30(0.02–4.1)	0.37
Bugoigo Village	1.08 (0.3–3.9)	0.99	6.86 (0.7–62.4)	0.08	3.56 (1.2–12.5)	**0.02**	9.7(2.3–41.2)	**0.002**
**Heavy intensity ( >300 µg/g)**								
3–5 years	1.43 (0.4–4.5)	0.59	1.71 (0.4–6.7)	0.44	1.7 (0.6–4.9)	0.23	0.41(0.1–1.2)	0.12
*Schistosoma mansoni*	1.25 (0.4–4.5)	0.79	0.91 (0.1–5.8)	0.92	0.85 (0.21–2.7)	0.99	1.09(0.2–4.7)	0.90
CCA	1.53 (0.8–2.9)	0.19	1.89 (0.8–4.5)	0.15	0.89 (0.6–1.3)	0.55	0.95(0.6–1.6)	0.86
FOB	0.84 (0.08–4.4)	0.99	1.12 (0.1–8.5)	0.90	2.9 (0.4–18.8)	0.17	1.42(0.2–9.6)	0.71
Bugoigo Village	1.79 (0.6–5.8)	0.30	3.67 (0.7–18.6)	0.11	7.58 (2.2–33.2)	**0.0001**	9.6 (2.7–34.0)	**0.0004**

All models were adjusted for age, sex, *Schistosoma mansoni* infection by faecal egg count ^+^ and urine cathodic circulating antigen (CCA), anaemia, malaria infection, fecal occult blood (FOB)^$^ and village.

Four different models were constructed after bivariate exploration of the possible associations between fecal calprotectin and *S. mansoni* infection. When calprotectin positivity (>50 µg/g) was considered as a binomial outcome, an association was found at baseline between CCA test positivity, younger age (3–5 years old) and residency in Bugoigo village. These associations, except for the village effect, were not seen 24 days after PZQ treatment.

When stratified by calprotectin intensity, ‘Medium’ intensity (150–299 µg/g) strongly correlated with egg-patent *S. mansoni* infection at baseline (OR: 12.5, *P* = 0.05) and follow up (OR: 6.8, *P* = 0.02), as well as with FOB positivity at baseline (OR: 7.5, *P* = 0.05). Before PZQ treatment, younger children (3–5 years old) had significantly increased levels of ‘Light’ intensity (51–149 µg/g) fecal calprotectin (OR: 2.4, *P* = 0.03) as well as increased ‘Medium’ intensity calprotectin at follow up (OR: 4.1, *P* = 0.03). FOB at follow up was associated with ‘Light’ intensity calprotectin in unadjusted analysis (Crude OR: 3.89, *P* = 0.05) although this was not seen when controlled for potential confounders. Across all intensities, residents of Bugoigo village were more likely to have positive fecal calprotectin.

## Discussion

In the context of performance of PZQ PC in targeted areas for control of morbidity associated with schistosomiasis , there is a growing need for standardized, affordable and non-invasive point-of-care tests as surrogate markers of disease [4]. Our results highlight the feasibility and reliability of both FOB and calprotectin assays as appropriate field-applicable correlates for intestinal schistosomiasis in young children and could be used to monitor morbidity in pre- and post-treatment settings. A positive test may also encourage people to partake in treatment campaigns as there is a generic need for better health advocacy.

Egg-patent schistosomiasis is highly prevalent in our study area despite repeated PZQ rounds in the past five years [17]. Indeed transmission here is known to be intense and the regional hot spot of disease for over 30 years [17]. We found that infection significantly correlates with markers of inflammation (calprotectin) and mucosal bleeding (FOB) and that this had a direct relationship with intensity of infection. Hence we can infer that intestinal schistosomiasis is also highly prevalent in the area and its public health implications remain to be fully appreciated. This is a rather worrisome result for an area with yearly anti-parasitic treatment campaigns and may be attributed at least in part, to the non-inclusion in the programs of pre-school age children that are known to harbour egg-patent infections [29], [30]. This leaves a large proportion of the infected population untreated despite recent WHO recommendations to include the under-fives in schistosomiasis PC programs [29],[23],[31]. Different tools for the detection of intestinal morbidity have been tested in different community surveys with mixed results [4]. In Uganda, a longitudinal study measuring the abdominal circumference ratio (ACR) in children with distended abdomens showed the widespread occurrence of a diseased state although with a weak association with morbidity-related egg-patent schistosomiasis [32]. Abdominal ultrasounds are widely used for hepatic and urinary schistosomiasis in areas where *S. mansoni* and *S. haematobium* are endemic, but they have little value for intestinal disease detection [33]. Even in a highly endemic *S. mansoni* area, hepatic and intestinal schistosomiasis may or may not co-exist and this emphasizes the importance of differentiating both clinical entities.

As a limitation to our study we could not compare the point-of-care tests that we used with colorectal endoscopy, the gold-standard diagnostic test for intestinal morbidity, however calprotectin has been widely previously validated in other studies as an acceptable inflammatory marker against colonoscopy [19], [21], [28]. Our proxy for morbidity was egg-patent *S. mansoni* infection. Infection is different from clinical disease and particularly in schistosomiasis egg-shedding may decrease with time and treatment, but organ damage from chronic inflammatory response to tissue trapped eggs is likely to persist. The association between FOB and *Schistosoma* infection has been clearly demonstrated before and it can therefore be considered a good surrogate marker for intestinal pathology [7]–[9], [15]. The advantages of the FOB immunochemical test are their relatively low cost ($0.6/test), easy to use with very little technical expertise required. All tests were reliable with no tests failing making the invalid test rate negligble.

The associations between Walukuba village and FOB, as well as between Bugoigo village and calprotection, are intriguing. The precise disease manifestation associated with a parasitic infection depends on a complex interplay between the host and parasite. In recent work, we did not find significant genetic differentiation between *S. mansoni* parasite populations from the three villages included in the present study (Betson et al., under review). However, there are tribal differences in the human populations between the three villages with certain tribes more or less represented in each village, for example most people in Walukuba belong to the Alur tribe, whereas in Bugoigo and Piida, the Banyoro and Bugungu tribes are also well-represented. These differences in local ethnography may least in part explain the associations between specific morbidity markers and particular villages.

The intertwined relationship between anemia and schistosomiasis is found across species and even in individuals with light parasitic loads [34],[35]. What remains to be elucidated, however, is the attributable fraction of intestinal losses versus other pathogenic mechanisms such as anemia of inflammation [36]. The burden of anemia in endemic communities leads to poor school and physical performance, such manifestations that will spiral down to an overall decreased quality of life and adult attainment [37], [38]. We found an interesting positive association between FOB and anemia at baseline, even after controlling for malaria co-infection. This suggests significant amounts of blood loss per rectum which would be consistent with intestinal schistosomiasis with high parasitic loads as reported in *S. japonicum* endemic settings [7]. The potential confounding effects of other parasitic infections known to cause intestinal bleeding (i.e. hookworms) was not of concern in our study site since none were detected. However hookworm should be considered in other endemic areas, e.g. in the Lake Victoria setting [16]. Due to mucosal inflammation and possibly bleeding polyps this presentation would require active clinical intervention [6]. At follow up, we documented a rapid response to PZQ with decreased overall FOB positivity in children with *S.mansoni* at baseline and no association between FOB and anemia. This is highly suggestive of effective mucosal healing.

Fecal calprotectin proved useful as an inflammatory marker in correlation with *S. mansoni* infection. It is important to note that the calprotectin range most significantly associated with any intensity *S. mansoni* infection (150–299 µg/g), is within the inflammatory bowel disease detection range and would warrant further endoscopic work up in industrialized countries [19], [28]. This is a significant finding since not all intestinal schistosomiasis is thought to be inflammatory in nature and therefore calprotectin detection could aid in identifying more active cases when used as a disease *severity* marker [5], [39]. The significant decline in calprotectin levels after PZQ treatment in children with egg patent *S.mansoni* at baseline, also suggests a positive response to anti-parasitic treatment, strengthening the relationship of the association calprotectin-schistosomiasis. Young children (<4 years of age) are known to have higher normal range values of fecal calprotectin. Regardless of this, the association found in our study between young age and high calprotectin levels is above the range of physiological parameters for healthy African children and warrants further investigation [40]. This will help define the inflammation attributable to schistosomiasis in infected children. For now, calprotectin is an expensive POC test ($10/test) and its widespread implementation in PC efforts could be restrained as a consequence unless outside international donor support was able to subsidise and reduce substantially the cost per test. Nevertheless, it provides very valuable clinical information and it should be considered useful to monitor intestinal disease, especially within a detailed future research study.

For the purpose of this study, we were able to ascertain a strong relationship between FOB, calprotectin and moderate and heavy egg intensities of *S. mansoni* infection. Even though the number of positive individuals decreased after PZQ treatment, there was no overall statistical significance after 24 days. However, when only children that were *S. mansoni* egg positive at baseline were analysed, both FOB and calprotectin did have a significant decrease and therefore detect an ameloriation response to PZQ treatment. This indeed suggests a short term mucosal healing response. A longer follow up would be needed to assess longer term responses. e.g. 2–3 month period.

What remains to be explored in a larger study, is the correlation between the different stages of mucosal involvement comparing POC assays with endoscopy before and after PZQ administration. This would allow for a clinical staging algorithm for intestinal schistosomiasis that could guide future community based treatment strategies. Due to its low-cost and ease of use, we advocate for the immediate integration of FOB as a morbidity monitoring tool in PC campaigns.
